# Correlation between Microalbuminuria and Hypertension in Type 2 Diabetic Patients

**DOI:** 10.12669/pjms.303.5042

**Published:** 2014

**Authors:** Alia Ali, Azeem Taj, Muhammad Joher Amin, Farrukh Iqbal, Zafar Iqbal

**Affiliations:** 1 Dr. Alia Ali, FCPS, Senior Registrar, Department of Medicine, Shaikh Zayed Postgraduate Medical Institute, Lahore, Pakistan.; 2 Dr. Azeem Taj, FCPS, Associate Professor, Department of Medicine, Shaikh Zayed Postgraduate Medical Institute, Lahore, Pakistan.; 3 Dr. Muhammad Joher Amin, FCPS, Assistant Professor of Gastroenterology, Shaikh Zayed Postgraduate Medical Institute, Lahore, Pakistan.; 4 Prof. Farrukh Iqbal, FRCP, Professor, Department of Medicine, Shaikh Zayed Postgraduate Medical Institute, Lahore, Pakistan.; 5 Prof. Zafar Iqbal, FRCP, Professor & Chairman, Department of Medicine, Shaikh Zayed Postgraduate Medical Institute, Lahore, Pakistan.

**Keywords:** Diabetic kidney disease, Hypertension, Microalbuminuria, Type 2 Diabetes Mellitus

## Abstract

***Background: ***Hypertension is commonly found in patients with Diabetic Kidney Disease (DKD). Microalbuminuria is the first clinical sign of involvement of kidneys in patients with type 2 diabetes. Uncontrolled hypertension induces a higher risk of cardiovascular events, including death, increasing proteinuria and progression to kidney disease.

***Objectives: ***To determine the correlation between microalbuminuria and hypertension and their association with other risk factors in type 2 diabetic patients.

***Methods: ***One hundred and thirteen type 2 diabetic patients attending the diabetic clinic of Shaikh Zayed Postgraduate Medical Institute, Lahore, Pakistan were screened for microalbuminuria and raised blood pressure. The study was conducted from November 2012 to June 2013.

***Results: ***Patients were divided into two groups. Group 1, those with normoalbuminuria (n=63) and Group 2, those having microalbuminuria (n=50). Group 2 patients showed higher blood pressure values as compared to Group 1. The results were statistically significant and showed poor glycemic control as a contributing risk factor.

***Conclusion: ***The study concluded that there is high frequency of hypertension among type 2 diabetics but still much higher among those having microalbuminuria. So, early recognition of renal dysfunction through detection of microalbuminuria and to start treatment without any delay will confer future protection from end stage renal disease as well as hypertension and its complications in type 2 diabetic patients.

## INTRODUCTION

Diabetes Mellitus remains a tremendous challenge to public health worldwide. Diabetes and Hypertension are common diseases that coexist at a greater frequency than chance alone would predict. Hypertension in the diabetic individual markedly increases the risk and accelerates the course of cardiac disease, peripheral vascular disease, stroke, retinopathy, and nephropathy.^1^ Type 2 Diabetic patients may be hypertensive for years prior to the onset of overt diabetes. At the time of diagnosis of type 2 Diabetes Mellitus, hypertension is found in approximately 70-80% of patients. Still blood pressure rises further in those patients who subsequently develop diabetic nephropathy.^2^

Diabetic nephropathy is the leading cause of end stage renal disease (ESRD) worldwide.^3^ It also accounts for a third of all patients requiring renal replacement therapies, which are prohibitively expensive and not widely available in our country due to cost and lack of expertise. The development of diabetic nephropathy is characterized by a progressive increase in the excretion of protein, particularly albumin, an early and continuing rise in systolic blood pressure, and a late decline in glomerular filtration rate, leading eventually to end stage renal failure. Systolic blood pressure may be particularly important and in the UKPDS (United Kingdom Prospective Diabetes Study), higher blood pressure was associated with a higher risk of macro vascular and micro vascular disease.^4^

Hypertension and Microalbuminuria commonly coexist. The mechanism is controversial but is thought to be a renal manifestation of generalized vascular endothelial dysfunction and strongly linked with increased cardiovascular risk. It is well appreciated both that coexisting hypertension exacerbates diabetic nephropathy and that diabetic nephropathy somehow results in a markedly increased risk of hypertension.

The American Diabetes Association recommends that patients with type 2 diabetes be tested for albuminuria at the time of initial diabetes diagnosis and yearly thereafter.^5^ Screening for the earliest stages of renal damage and aggressively controlling blood pressure can help prevent more severe renal involvement. Blood pressure control is at least as important as glucose control, especially after the onset of renal damage.^6^

The present study was initiated to see the correlation between microalbuminuria and hypertension in type 2 diabetics and its association with other risk factors.

## METHODS

A total of one hundred and thirteen type 2 diabetic patients who attended the diabetic outdoor clinic of Shaikh Zayed Postgraduate Medical Institute (SZPGMI), Lahore, Pakistan were included in the study.

The study was conducted over a period of eight months. Patient’s age ranges from 30-70 years. Informed consent was obtained from each patient. A structured questionnaire regarding the demographic data such as age, gender, height, body weight while wearing light weight clothing, without shoes and family history of DM.

Blood pressure, smoking habit were recorded for each patient. Diabetic patients suffering from any other medical problem were excluded from the study. Co-morbidities like chronic heart disease, renal and liver diseases as evidenced by either history of ischemic heart disease or alteration in ECG; history of renal disease or disturbed BUN creatinine; history of liver disease such as hepatitis B or C positive or disturbed liver function tests were excluded from the study.

BMI was calculated as weight (Kg) divided by height (m^2^). Blood pressure was measured with a suitable mercury sphygmomanometer. Readings were taken after the patients had been resting comfortably, back supported in the sitting or supine position, for at least 5 minutes and at least 30 minutes after smoking or coffee ingestion, by a physician or a trained nurse. Blood pressure was measured two times at 5 minutes interval. The 1^st^ and the 5^th^ Korotokoff’s sound were used to determine the systolic (SBP) and diastolic (DBP) measurements respectively. The second blood pressure measurement was used as the blood pressure for the individual. Mean arterial pressure (MAP) was calculated as systolic + (2xdiastolic) pressure.

High blood pressure was defined as systolic blood pressure (SBP) ≥140mmhg and/ or diastolic blood pressure (DBP) of ≥90mmhg or use of anti-hypertensive medications.^7^ Blood samples were obtained for HbA1c test that was estimated using chromatographic method by antiticalcon kit from USA. All assays were performed as per instructions of the manufacturers.

HbA1c was assessed by mean of three consecutive Hba1c levels, either <6.5% or ≥6.5%. Those patient were included who had no evidence of proteinuria in urinalysis and without abnormal serum BUN and creatinine.

Morning mid-stream urine sample, negative for proteinuria by Albustix was used to calculate micro albumin: creatinine ratio in mg/g. Micro albumin was carried out using ELISA assay developed at NHRC and validated at NETRIA UK, whereas creatinine was done by calorimetric methods using Fortress kit USA. If the ratio was <30mg/g the patient was normoalbuminuric, ratios between 30-300mg/g were indicative of microalbuminuria and above 300mg/g revealed macroalbuminuria.

Subjects were excluded from the study if they came to the clinic after vigorous exercise, had any serious illness such as history of heart failure, UTI or were known patients of nephropathy.

Statistical analysis was done using SPSS version 20. Descriptive statistics were compared for continuous variables, and percentages for categorical variables. Association between outcome variables and independent variables were sought using T-Test.

## RESULTS

Patients were divided into two groups as assessed by microalbuminuria calculated as micro albumin: creatinine ratio in mg/g.

Group 1: (n=63) were those patients with micro albumin: creatinine ratio <30mg/g.

Group 2: (n=50) were those patients with micro albumin: creatinine ratio between 30-300mg/g.

There were 16 males and 47 females in group 1 with a mean age of 49.89 ±9.34 in years while in group 2 there were 23 males and 27 females with a mean age of 48.4 ±9.35 in years.

***Baseline Characteristics of two groups:***

Positive family history of DM was in 63.49% in group 1 and 78.6% in group 2 respectively. Mean BMI in group 1 was 28.90 ±4.17 while in group 2 it was 28.61 ±4.09. These results showed no significant statistical difference (Table-I). Mean HbA1c in group 1 was 76.25 ±16.99 while in group 2 it was 80.36 ±8.24. These results were statistically significant (Table-I). Group 2 patients showed higher BMI as compared to group 1 patients (p<0.001).

**Table-I:** Baseline Characteristics of Two Groups

*Variables*	*Group 1(n=63)*	*Group 2(n=50)*	*P Value*
Gender (n=113) Males = 39 Females = 50	16(41.02%) 47(63.51%)	23(58.97%) 27(36.48%)	
Age (years)	Mean=49.89 ± 9.34	Mean=48.4 ± 9.35	.860
Family History of DM Positive Negative	40(63.49%) 23(36.50%)	36(72%) 14(28%)	
BMI	Mean=28.90± 4.17	Mean=28.61 ±4.09	.707
HbA1c	Mean=7.31 ±1.59	Mean=7.72±.807	<.001

The two groups were also assessed for difference of blood pressure that included systolic (SBP), diastolic (DBP) and mean arterial pressure (MAP). Group 2 showed mean systolic pressure (MSP) higher than group 1 as evidenced by p<0.001 that is statistically significant. Similarly group 2 showed mean diastolic pressure (MDP) and mean arterial pressure (MAP) higher than group 1 as evidenced by p<0.001 that is again statistically significant. These results showed significant statistical difference (p value=<.001). Group 2 patients showed higher blood pressure values as compared to group 1 patients.

**Table-II:** Difference of Blood Pressures between Two Groups

*Variables*	*Group 1 (n=63)*	*Group 2 (n=50)*	*P Value*
Systolic Blood Pressure	Mean = 133.4 ±15.92	Mean = 150.12 ±5.47	<.001
Diastolic Blood Pressure	Mean = 87.54 ±7.77	Mean = 94.20 ±3.95	<.001
Mean Blood Pressure	Mean =102.86 ±10.12	Mean =112.89 ±3.69	<.001

## DISCUSSION

Over the past decades, there has been a significant worldwide increase in the incidence of diabetes mellitus.^8^ This current global epidemic is associated with an increase of cardiovascular diseases that primarily accounts for the increase in morbidity and mortality seen in patients with diabetes.^9^^,^^10^ The high prevalence of microvascular complications of diabetes such as diabetic nephropathy means that the number of patients with end stage renal disease due to diabetes will also increase dramatically.^11^ Hence diabetes especially type 2 diabetes is becoming the main reason for patients to start renal replacement therapy.^12^^,^^13^

United Kingdom prospective diabetes study (UKPDS) a well-known study on diabetes mellitus showed that the presence of hypertension is a risk factor for microalbuminuria and retinopathy and that reducing the incidence of chronic complications was significantly associated with the amplitude of systolic blood pressure decrease, the lowest risk corresponding to a systolic blood pressure below 120mmHg.^4^

The risk factors associated with microalbuminuria were found to be poor glycemic control and hypertension. It is now widely appreciated that the excretion of even small amounts of albumin in the urine may portend serious future events, such as elevation of systemic arterial pressure, cardiovascular disease and progressive renal dysfunction.^14^^,^^15^ In general microalbuminuria is a sensitive marker for damage induced by diabetes.^16^

The combination of hypertension and diabetes is an especially dangerous clinical situation, both for risk of macro vascular and microvascualr complications of diabetes and for diabetes related and overall mortality.^6^ Hypertension is unfortunately also very common in patients with diabetes. Hypertension occurs in 50% of patients with Diabetes and results in a sevenfold increase in mortality.^17^ Concomitant nephropathy in patients with diabetes and hypertension results in a 37-fold increase in mortality. Hypertension is probably both a cause and an effect of diabetic nephropathy.^18^

Studies have shown an association between microalbuminuria and high levels of blood pressure. Ahmedani et al. showed microalbuminuria positive group had a higher systolic and diastolic blood pressure compared to microalbuminuria negative group (p<0.001) which has been observed by others.^19^ Similarly Arkedani et al. and Varghese et al. reported a good statistical correlation between the prevalence of microalbuminuria and the diastolic blood pressure.^20^^,^^21^ Pasko et al found that microalbuminuric patients had higher systolic and diastolic blood pressure, suggesting that systolic blood pressure is a significant risk factor for diabetic nephropathy.^22^ Svenson et al. showed that high blood pressure increased the risk of developing signs of nephropathy.^23^ Our study also found a significant statistical correlation between hypertension and microalbuminuria and this finding was also strongly associated with poor glycemic control.

Hypertension can cause microalbuminuria and hypertensive nephropathy that can accelerate the progression of diabetic nephropathy. Thus hypertension is probably both a cause and an effect of diabetic nephropathy. Epidemiological studies have shown that identifying and monitoring patients with microalbuminuria is important because its treatment can prevent or postpone overt nephropathy.^17^^,^^24^ Higher values of Systolic blood pressure and diastolic blood pressure in microalbuminuric than in normoalbuminuric patients suggests that hypertension is associated with microalbuminuria.^25^

## CONCLUSION

High prevalence of microalbuminuria in diabetic patients and its positive association with blood pressure suggests that screening for microalbuminuria is essential for intervention and prevention of further complications like end stage renal disease and cardiovascular disease. Once overt nephropathy is present, progression cannot be halted, only slowed. It is much more effective to screen for early nephropathy with sensitive tests for microalbuminuria and to prevent or halt the earliest stages of damage by vigorous control of hypertension and hyperglycemia.

## Authors contributions:

**FI, AT, AA:** Conceived, designed, and did statistical analysis and editing of manuscript

**AA, MJA**: Did data collection and manuscript writing.

**ZI**: Did review and final approval of manuscript.
